# The impact of poor medication knowledge on health-related quality of life in people with Parkinson’s disease: a mediation analysis

**DOI:** 10.1007/s11136-021-03024-8

**Published:** 2021-11-19

**Authors:** Hannah M. Zipprich, Sarah Mendorf, Aline Schönenberg, Tino Prell

**Affiliations:** 1 Present Address: Department of Neurology, Jena University Hospital, Am Klinikum 1, 07747 Jena, Germany; 2 Center for Healthy Ageing, Jena University Hospital, Jena, Germany

**Keywords:** Medication adherence, Parkinson’s disease, Mediation analysis, German Stendal Adherence with Medication Score, Depression, Patient outcome assessment

## Abstract

**Purpose:**

This study aimed to determine how limited medication knowledge as one aspect of health literacy contributes to poorer health-related quality of life (HRQoL) in people with Parkinson’s disease (PD).

**Methods:**

Demographical data, PD-specific data (MDS-Unified Parkinson’s Disease-Rating Scale, Nonmotor symptom scale), and data about depressive symptoms (Beck’s depression inventory), cognition (Montreal cognitive assessment), HRQoL (Short-Form Health Questionnaire-36, SF-36), and medication knowledge (names, time of taking, indication, dosage) were assessed in 193 patients with PD. Multivariate analysis of variance (MANOVA), multivariate analysis of covariance, and mediation analyses were used to study the relationship between medication knowledge and HRQoL in combination with different mediators and covariates.

**Results:**

Overall, 43.5% patients showed deficits in at least one of the 4 knowledge items, which was associated with higher age, number of medications per day and depression level, and poorer cognitive function, motor function, and lower education level. Using one-way MANOVA, we identified that medication knowledge significantly impacts physical functioning, social functioning, role limitations due to physical problems, and role limitations due to emotional problems. Mediation models using age, education level, and gender as covariates showed that the relationship between knowledge and SF-36 domains was fully mediated by Beck’s Depression Inventory but not by Montreal Cognitive Assessment.

**Conclusions:**

Patients who expressed unawareness of their medication did not necessarily have cognitive deficits; however, depressive symptoms may instead be present. This concomitant depressive symptomatology is crucial in explaining the contribution of nonadherence and decreased medication knowledge to poor quality of life.

**Supplementary Information:**

The online version contains supplementary material available at 10.1007/s11136-021-03024-8.

## Introduction

Knowledge regarding one’s prescribed medication (in the following called medication knowledge) is essential in chronic disorders such as Parkinson’s disease (PD). PD is a common and chronic neurodegenerative disorder characterized by motor symptoms and a plethora of nonmotor symptoms. Motor symptoms usually include slowness of movements, rigidity, or tremor. Common nonmotor symptoms are depression, cognitive deficits, and dementia which all impact independence, activities of daily living, and health-related quality of life (HRQoL) [1].

HRQoL plays a major role especially in treatment for chronic diseases such as PD [2]. HRQoL itself encompasses various physical, social, and mental functioning abilities and includes relationships, perceptions, and satisfaction [3]. As the disease progresses, the medications usually have to be constantly adapted to the needs of the patient. This often means that many different medications have to be taken to control and manage PD symptoms. Therefore, the issue of adherence is of enormous importance for this patient group. Adherence is described as the extent to which a person's behaviors correspond to the agreed recommendations from their healthcare provider [4]. However, there are various reasons as to why people do not or cannot follow the instructions they are given for prescribed treatments. This nonadherence can cause a significant deterioration in mobility and HRQoL in PD [5–8]. Systematically, the factors associated with nonadherence to medications can be divided into patient characteristics, disease-related factors, financial/economic and health system barriers, patient–provider relationship factors, and treatment-related factors [9–11]. In PD in particular, many factors were found to be associated with nonadherence, such as education level, marital status, depression, cognition, and regimen complexity [8, 9, 12–14]. Another important aspect of nonadherence is the knowledge about the prescribed medication [6, 8]. This means, for example that patients know why they should take a certain medication or when and how they should take it.

The medication knowledge is furthermore closely linked to the construct of health literacy. Health literacy, defined as “the degree to which individuals have the capacity to obtain, process, and understand basic health information and services needed to make appropriate health decisions” [15] and has been associated with medication knowledge, which in turn is an important factor associated with better medication adherence [16, 17]. In contrast, patients with insufficient medication knowledge tend to misunderstand the physicians’ recommendations and may intentionally or unintentionally (i.e., by forgetting to take their medication) modify the dosage of their prescribed medication [18, 19]. Moreover, nonadherence and poor health literacy have been found to potentially contribute to worsening motor function and poor HRQoL [20].

In summary, medication knowledge is closely linked to health literacy and lower levels of nonadherence and may, therefore, have an impact on HRQoL. However, it remains so far unclear how medication knowledge exerts its effect on HRQoL. Therefore, with this study, we aimed to determine how limited medication knowledge (as one aspect of health literacy) contributes to poorer HRQoL in PD. Given that depression and cognitive dysfunction are common in PD and major predictors of lower HRQoL as well as nonadherence, we hypothesized that both depression and cognition could mediate the effect of medication knowledge on HRQoL.

## Methods

### Participants and assessments

This observational cross-sectional study was approved by the local ethics committee of the Jena University Hospital (5290–10/17). All subjects provided written-informed consent in accordance with the Declaration of Helsinki. Patients with PD who were treated at the Department of Neurology of the Jena University Hospital, Germany, from January 2019 until March 2020 were consecutively recruited, until enrollment had to be stopped due to the beginning of the COVD-19 pandemic. The inclusion criterion was PD diagnosis according to the diagnostic criteria of the Movement Disorder Society (MDS) (diagnosis was made by a movement disorder specialist TP), ability to understand and fulfill a questionnaire, partial or full self-management of medication. The exclusion criteria were delirium and severe dementia that hindered participants from understanding and completing a questionnaire. All tests were conducted when the patients were on medication. After excluding seven patients due to missing data, a total of 193 subjects were analyzed.

Data were collected by our trained research staff. After a short introduction to the aims and methods of the study, the Montreal Cognitive Assessment (MoCA) was administered, which is a screening tool to assess several components of cognition, including visuo-spatial and executive function, language, memory, attention, and orientation [21]. The MoCA is commonly used to detect cognitive impairments and has been validated for PD patients in particular [22]. Based on this face-to-face testing of cognition, we were able to include patients with MoCA scores < 21 points (common screening threshold for PD dementia if they reported self-management of medication and understood the study aim and questionnaires). The following demographic and clinical data were then collected age, gender, marital status (single, divorced, widowed, or married), level of education (high, German Abitur or University; medium, German Realschule or General Certificate of Secondary Education; low, German Hauptschule or no school), and total daily number of medications administered in any pharmaceutical form. Beck’s depression inventory II (BDI), a 21-item self-report questionnaire assessing depressive symptoms on a scale from 0 to 3, was used to assess depression level based on the cumulative score with higher scores indicating higher levels of depression. The BDI is a psychometrically sound and valid instrument that has been previously validated for use with PD patients [23, 24]. To assess PD-specific symptoms, the MDS-sponsored revision of the Unified Parkinson’s Disease-Rating Scale III (MDS-UPDRS III), a comprehensive screening performed by trained medical staff [25] was used to assess severity of motor symptoms such as speech, rigidity, tremor, gait, and other movements on a scale from each 0 to 4, with higher scores indicating more severe pathology. The revised nonmotor symptoms questionnaire (NMS-Quest), which is a 30-item screening instrument to capture symptoms such as fatigue or pain [26], was used to detect the presence of PD-typical nonmotor symptoms.

The Short-Form Health Questionnaire (SF-36) was administered for assessments of HRQoL [27]. It includes 36 questions with normalized scores ranging from 0 (absence of HRQoL) to 100 (optimal HRQoL) across eight dimensions: physical functioning, role limitations due to physical health, bodily pain, general health, vitality, social functioning, role limitations due to emotional problems, and emotional well-being. The SF-36 is psychometrically sound and is recommended for measuring HRQoL in patients with PD, with high correlations having been found between SF-36 and similar scales on disease-specific PDQ-39 [28, 29]. Given the lack of evidence supporting the physical and mental health components in PD, the scores of these two components were not used in our study [29].

Medication knowledge was determined using the following questions derived from the German Stendal Adherence with Medication Score (SAMS) [30, 31]. The SAMS includes 18 questions forming a cumulative scale (0–72), in which 0 indicates complete adherence and 72 indicates complete nonadherence [31, 32]. Responses were scored on the following Likert scale: for all (0), most (1), half (2), some (3), and none (4).

- Do you know the reason for taking your medication?
- Do you know the dosages of your medication?
- Are you familiar with the timing of taking the medication?
- Do you know the names of the medications you are taking?

For each patient, the scores for each question were summed, with high values indicating poor knowledge and low values indicating better medication knowledge.

### Statistical analysis

The SPSS statistical computer package (version 25.0; IBM Corporation, USA) was used for all statistical analyses. Values are presented as means and standard deviations or medians and interquartile ranges. Categorical variables are presented as numbers or percentages. For all analyses, a *p* value of < 0.05 indicated statistical significance.

First, we described the cohort using descriptive statistics. Normal distribution was determined using the Shapiro–Wilk test. Patients with good and poor knowledge were compared with demographic data using the Student’s *t* test or *U* test. Linear regression with backward selection was used to determine the association between knowledge score and clinical variables that were identified as significant (*p* < 0.01) during univariate analyses (after exclusion of multicollinearity and autocorrelation).

Multivariate analysis of variance (MANOVA) was used to study the effects of knowledge level on SF-36 domains. Correlations between dependent variables were low (*r* < 0.90), indicating that multicollinearity was not a confounding factor in the analysis. Two multivariate outliers were found using the Mahalanobis distance (*p* > 0.001) and excluded from analysis. The presence or absence of outliers did not affect the results. Homogeneity of the error variances was observed for seven SF-36 domains (*p* > 0.05) but not for role limitations due to physical health as assessed using Levene’s test. Homogeneity of covariances was noted as assessed using Box’s test. Multivariate analysis of covariance (MANCOVA) was used to adjust these findings for BDI and MoCA.

To understand the underlying mechanisms explaining the relationship between medication knowledge and HRQoL, a mediation model was used [33]. A mediator is a variable that accounts for the relationship between a predictor and an outcome (here, different levels of medication knowledge and HRQoL, respectively) and may fully (full mediation) or partially (partial mediation) account for the relationship. Therefore, a mediator helps explain the presence of a relationship between the two variables. Mediation analyses were performed using the PROCESS macro by Hayes (2018) that utilizes ordinary least-squares regression, yielding unstandardized path coefficients for total, direct, and indirect effects [34]. BDI and/or MoCA were used as moderator(s) of the relationship between knowledge score and SF-36 domains, whereas age, education level, and sex were entered as covariates. The statistical significance of the direct and indirect effects was evaluated using 10,000 bootstrap samples to create bias-corrected confidence intervals (CIs; 95%). The relationship between all variables involved in the mediation analysis was approximately linear, as assessed using visual inspection of the scatterplots after LOESS smoothing. Given that the pure effect of mediation is described by the indirect effect, this is the most important criterion for mediation regardless of the other prerequisites [35, 36].

Because we also included people with cognitive deficits (who might fill out questionnaires less validly), we determined validity of HRQoL report in people with and without cognitive deficits. Internal consistency between the SF-36 subdomains was evaluated using Cronbach’s coefficient α where values of > 0.70 indicated adequate internal consistency [37]. Floor and ceiling effects were defined as the proportion of respondents scoring the highest (ceiling) or lowest (floor) possible score across any given domain. These effects were considered present if at least 15% of respondents reached the lowest or highest possible score, respectively [37]. Convergent validity was measured by calculating the Spearman correlation coefficient of all SF-36 subdomain scores with BDI.

## Results

### Predictors of poor medication knowledge

**Fig. 1:**
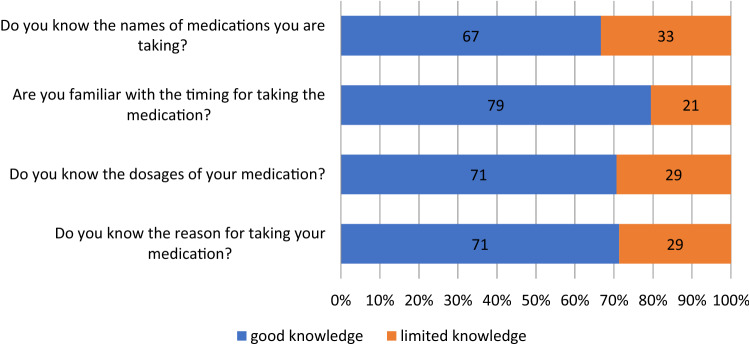
Percentage of knowledge questions indicating good (item value = 0) and limited knowledge (item value > 0)

Detailed clinical data from the 193 subjects are summarized in Table 1, and the pattern of medication knowledge is presented in Fig. 1. Patients with limited medication knowledge (43.5%) were older, took more medications per day, had poorer cognitive and worse motor functions (higher MDS-UPDRS III), higher levels of depression, and lower education levels compared with patients with good medication knowledge (Table 1). Linear regression analysis found that impaired knowledge was associated with higher age (*β* = 0.16, *p* = 0.07), higher number of medications per day (*β* = 0.22, *p* = 0.007), BDI (*β* = 0.19, *p* = 0.016), and MoCA (*c* =  − 0.20, *p* = 0.025) [*F*(4, 134) = 9.25, corrected *R*^2^ = 0.19, Durbin Watson = 2.2].

**Table 1:** Characteristics of the cohort

	Entire cohort		Good knowledge		Limited knowledge		*p* value*
	*N*	%	*N*	%	*N*	%	
Sex							
Female	75	38.9	47	43.1	28	33.3	0.167
Male	118	61.1	62	56.9	56	66.7	
Marital status							
Married	143	75.7	83	77.6	60	73.2	0.476
Widowed, divorced	40	21.2	22	20.6	18	22.0	
Single	6	3.2	2	1.9	4	4.9	
Education level							
Low	42	22.1	16	15.1	26	31.0	0.027
Middle	61	32.1	39	36.8	22	26.2	
High	87	45.8	51	48.1	36	42.9	
	M	SD	M	SD	M	SD	
Age	71.2	8.2	69.6	8.2	73.1	8.0	0.004
Number of medications per day	7.0	4.3	5.5	3.4	8.9	4.5	< 0.001
MDS-UPDRS III	25.5	14.1	22.4	12.5	28.8	15.1	0.008
Disease duration (years)	9.5	5.8	9.3	5.7	9.7	6.1	0.975
BDI	11.3	7.0	10.2	6.7	12.9	7.2	0.010
NMS-Q	10.3	5.0	9.6	4.9	11.0	5.1	0.099
MoCA	22.6	4.2	23.5	3.6	21.3	4.5	< 0.001

^*^Refers to group comparison between individuals with good and limited knowledge

*MoCA* Montreal Cognitive Assessment; *BDI* Beck’s depression inventory II; *MDS-UPDRS III* the MDS-sponsored revision of the Unified Parkinson’s Disease-Rating Scale III, *NMS-Quest* revised nonmotor symptoms questionnaire

### Validity of self-reports with regard to cognitive state

Given that patients with MoCA scores below 21 points were also included, we analyzed the validity of the SF-36 to assess a potential impact of impaired cognition. For this purpose, the cohort was divided into patients with MoCA scores of ≥ 21 (*n* = 136, 70.5%) and < 21 (*n* = 57, 29.5%). For both MoCA groups, ceiling effects were present for social functioning, role limitations due to emotional problems, and pain subdomains, whereas floor effects were present for role limitations due to physical problems and role limitations due to emotional problems subdomains of the SF-36. No differences in internal consistency and convergent validity were found between both cognitive groups (Supplement Tables 1 and 2).

### Impact of poor medication knowledge on HRQoL

**Fig. 2:**
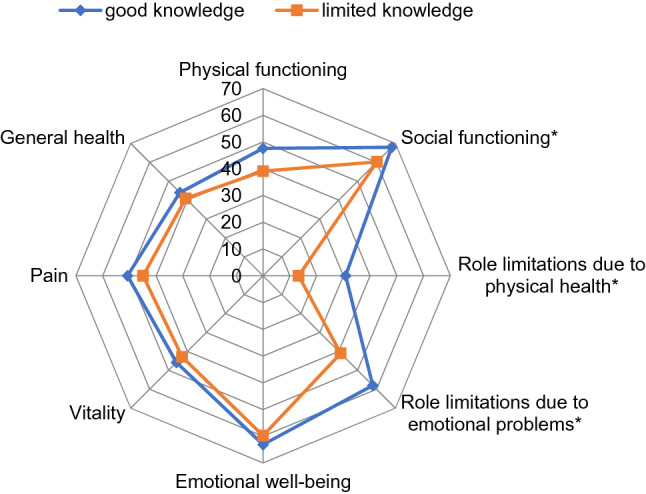
Polar plot indicating SF-36 domain scores in patients with good and limited medication knowledge. Higher scores represent better health and functioning. *p < 0.05

Univariate analyses showed that individuals with limited knowledge had lower scores for social functioning (*p* = 0.044), role limitations due to physical health (*p* = 0.004), and role limitations due to emotional problems (*p* = 0.017) compared with those with good knowledge (Fig. 2).

MANOVA was then performed to analyze the impact of knowledge level on the eight SF-36 domains. A one-way MANOVA revealed significant differences between knowledge level in the combined dependent variables [*F*(8, 170) = 2.09, *p* = 0.039, partial *η*^2^ = 0.09, Wilk’s Λ = 0.91]. Our results showed a significant difference between knowledge levels for physical functioning [*F*(1, 177) = 5.47, *p* = 0.020, partial *η*^2^ = 0.03], social functioning [*F*(1, 177) = 6.06, *p* = 0.015, partial *η*^2^ = 0.033], role limitations due to physical problems [*F*(1, 177) = 12.81, *p* < 0.001, partial *η*^2^ = 0.067], and role limitations due to emotional problems [*F*(1, 177) = 6.34, *p* = 0.013, partial *η*^2^ = 0.035].

MANCOVA was conducted to examine whether BDI and MoCA could account for these findings. Accordingly, our analysis found that BDI (Wilks’s *λ* = 0.63, *p* < 0.001, partial *η*^2^ = 0.37), and MoCA (Wilks’s *λ* = 0.87, *p* = 0.004, partial *η*^2^ = 0.12), but not knowledge level (*p* = 0.20) were significant in the model. We, therefore, hypothesized that the effect of knowledge level on HRQoL could be mediated through depression and/or cognitive function. To address this issue, a mediation analysis was performed.

### Mediation analyses

**Fig. 3:**
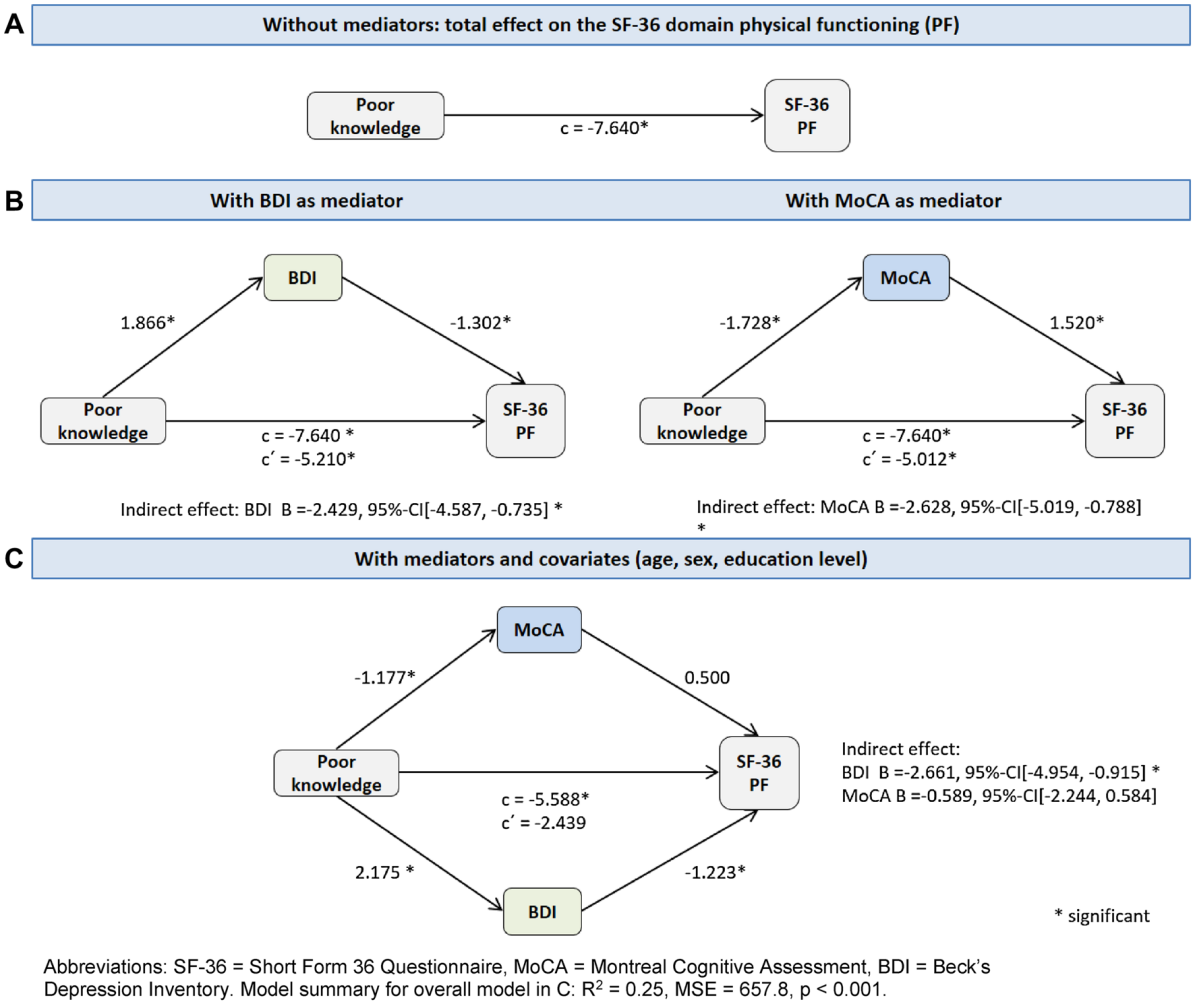
Mediation analyses: Mediation diagrams for the SF-36 domain Physical functioning. *SF-36* short-form 36 Questionnaire, *MoCA* Montreal Cognitive Assessment, *BDI* Beck’s Depression Inventory. Model summary for overall model in C: *R*^2^ = 0.25, MSE = 657.8, *p* < 0.001

Simple mediations were performed to analyze whether knowledge level is associated with the four SF-36 domains (physical functioning, social functioning, role limitations due to physical problems, and role limitations due to emotional problems) and whether an indirect effect would be mediated by the BDI and/or MoCA. The model calculation for the domain physical functioning is presented herein. We found that knowledge level had a direct effect on physical functioning (indicated by path c in Fig. 3A–C). This effect was partly mediated by depression or cognition, when only the BDI *or* MoCA was entered as one mediator into the model. This can be observed in Fig. 3B where coefficient c´ between knowledge level and physical functioning remains significant after entering the mediator (i.e., BDI or MoCA). After entering BDI *and* MoCA at the same time as mediators into the model, significant indirect effects were seen through both depression and cognition (BDI (B = 1.866, *p* = 0.004) and MoCA (B =  − 1.728, *p* < 0.001). Here, the association between knowledge level and physical functioning was fully mediated by BDI and MoCA [indirect effect for BDI *ab* =  − 2.352, 95% CI (− 4.456, − 0.708); for MoCA *ab* =  − 2.431, 95% CI (− 4.759, − 0.626)].

However, after adding age, education level, and gender as covariates, the relationship between knowledge level and physical functioning was fully mediated by the BDI but no longer by the MoCA [indirect effect for BDI *ab* =  − 2.661, 95% CI (− 4.918, − 0.892); for MoCA *ab* =  − 0.588, 95% CI (− 2.213, 0.619)] (Fig. 3C). Results for social functioning, role limitations due to physical problems, and role limitations due to emotional problems are presented in Supplement Fig. 1. Briefly, the influence of knowledge on these three domains was mediated through BDI but not MoCA (after correcting for age, education level, and sex).

## Discussion

The aim of this study was to understand how limited medication knowledge contributes to poorer HRQoL in people with Parkinson´s disease (PD), especially in relation to other important predictors of HRQoL such as depression and cognitive impairment. Overall, our findings showed that poorer medication knowledge was associated with higher age, more medications per day, poorer cognitive function, poorer motor function, and lower education level. MANOVA revealed that poorer medication knowledge was associated with poorer HRQoL in the SF-36 domains social functioning, physical function, role limitations due to physical health, and role limitations due to emotional problems. The social function domain describes the reduction of social activities such as activities with friends, whereas the physical function domain represents difficulties with activities of daily living (ADLs), such as body care. The domain of role limitations due to physical health includes difficulties with work or ADLs caused by physical health problems, such as gait disturbances, whereas the domain of role limitation due to emotional problems domain involves problems with work or ADLs caused by emotional problems, such as depression or fatigue [38]. Our mediation analysis revealed that the relationship between knowledge level and SF-36 domains was mediated by depression but not by cognition. Significant indirect effects can occur in the absence of significant total or direct effects, such as in the social function and role limitation due to emotional problems domains (Supplement Fig. 3A and C). Common to both scenarios is the finding that the lack of an effect, whether total or direct, does not preclude the possibility of observing indirect effects [35].

Our results show that the interplay between nonadherence and HRQoL can only be understood after considering cognition and depression in PD. This leads to some conclusions for clinical practice. Accordingly, being unaware of their medication does not necessarily depend on cognitive deficits but is instead also linked to depressive mood. This concomitant depressive symptomatology is ultimately crucial for explaining why nonadherence and decreased medication knowledge contribute to a poorer HRQoL, which is in turn associated with increased nonadherent behavior, such as intentional nonadherence (i.e., changing medications without consulting the physician). Depression has been shown to strongly influence HRQoL [39, 40]; however, the exact effect of depression is not yet fully understood, especially with regard to the complex association among worse health status, health literacy, and depression [41]. A previous study reported that, while depressed patients showed significantly worse health status, low health literacy itself did not predict depressive symptomology [42]. Thus, it is crucial to disentangle the exact aspects of depression and its related factors that influence HRQoL, especially with regard to different subfactors such as loss of interest, apathy/lack of motivation, and somatic symptoms [43, 44]. As it was the primary aim of the present analysis to understand how medication knowledge is related to HRQoL and which overall factors influence this relationship, the available data cannot provide information on the exact parameters of depressive mood that drive this influence. However, future studies are needed to differentially understand the detailed aspects of depressive mood related to medication knowledge and HRQoL.

The issue is further complicated by the fact that some overlap exists between depression and cognitive impairment [44, 45]. Depression, a common nonmotor symptom observed in patients with PD, can cause reversible cognitive impairment, particularly poorer concentration, impaired memory, and impaired problem solving, without the additional presence of dementia [46]. The association of cognition with nonadherence is complex and is yet to be fully understood. Some studies found that cognitive impairment is generally a major predictor of nonadherence [47], and the ADHESON study showed that patients with cognitive deterioration determined using a simple self-report adherence questionnaire (Morisky–Green test) were 2.1 times more likely to incorrectly administer treatment [17]. In contrast, a previous meta-analysis found no association between cognitive impairment and medication nonadherence in poststroke patients [48]. Likewise, our previous study observed no association between self-reported nonadherence and cognition in patients with PD [49].

Nonetheless, patients with cognitive deterioration need to be distinguished from those who cannot name their medication. This can be tested using the pill questionnaire wherein all medications and dosages are directly queried. Difficulties in the pill questionnaire can indicate cognitive impairment and dementia [50]. In contrast, the present study was a subjective assessment of the patients, and hence, we used a self-report questionnaire, which means that no statements can be made regarding objective knowledge (like in the pill questionnaire) about drugs. In particular, the presence of depression may impact how participants answer in self-reported questionnaire [51]. At this point, it is also of note to say that the measurement of nonadherence is complex and that the available instruments do not capture all aspects of this construct [52]. However, while the SAMS used in this study may not encompass all aspects of adherence, the key focus of the analysis was put on medication knowledge as a subfactor related to adherence.

The strength of the current study lies in the inclusion of patients with and without cognitive deficits, which better reflects real-world situations in elderly patients with PD. Given that cognitive deficits are highly prevalent among the elderly and patients with PD, our approach enhances the generalizability of these results. Although several studies have shown that self-reports are valid even in patients with dementia, we did prove the validity of our self-reported outcome measures. In particular, convergent validity was measured by calculating the Spearman correlation coefficient of all SF-36 subdomain scores with BDI. Here, no differences were found with regard to internal consistency and convergent validity between individuals with higher and lower MoCA. Our results were consistent with those presented in earlier studies including other cohorts, which showed that the BDI II total scores correlated strongest with SF-36 subdomains of mental health, vitality, and social functioning [53, 54]. We, therefore, conclude that the self-reports of our participants are valid and sound.

Our study has a few limitations. Studying patients with PD limits the generalizability of the results to other cohorts and diseases. Moreover, this explorative analysis could not account for all possible mediator variables, such as anxiety and personality traits. Furthermore, we focused on self-reported medication knowledge and did not objectify the knowledge by detailed interviews or specifically naming the prescribed medication. For a holistic understanding of the role of cognition and depression as mediators for medication knowledge, incorporating additional methods to assess the knowledge, such as using the pill questionnaire, would be useful.

## Conclusion

The present study showed that self-reported medication knowledge affects HRQoL and that this effect is mainly mediated by depression rather than by cognitive impairment. Therefore, people who claim not to know their medications are more likely to be depressed than cognitively impaired. This is important to remember, for example, when asking about knowledge of prescribed medications during an interview. Thus, special attention in the general care of patients with PD should be paid to depressive mood and cognitive deficits to improve knowledge about medication, adherence, and therefore, HRQoL. Simple adherence questionnaires can, therefore, help screen not only for people who have poor medication knowledge, but also for people who are at risk for poor HRQoL and depressive mood.

## Supplementary Information

Below is the link to the electronic supplementary material.

Supplementary file1 (DOCX 153 kb)

## Declarations

### Availability of data and material

Dataset is available from the corresponding author upon reasonable request for scientific purpose.
