# Supplementary material for: The impact of poor medication knowledge on health-related quality of life in people with Parkinson’s disease: a mediation analysis
Source: Qual Life Res. 2021 Nov 19;31(5):1473–82. doi: 10.1007/s11136-021-03024-8 (PMC9023397; doi:10.1007/s11136-021-03024-8)
Supplement: Supplementary file 1 — Supplementary file1 (DOCX 153 kb) [file 11136_2021_3024_MOESM1_ESM.docx]

Supplementary Materials

# **Supplement Table 1.** Convergent validity of the Short Form Health 36 questionnaire: Spearman correlation between SF-36 domains and Beck’s depression inventory II

| **SF-36 subdomains** | **MoCA <21** | | | **MoCA ≥ 21** | | |
| --- | --- | --- | --- | --- | --- | --- |
|  | **r** | ***P*** | **N** | **r** | ***P*** | **N** |
| Physical functioning | −0.401 | 0.002 | 57 | −0.324 | <0.001 | 135 |
| Social functioning | −0.582 | <0.001 | 57 | −0.400 | <0.001 | 134 |
| Role limitations due to physical problems | −0.413 | 0.002 | 52 | −0.328 | <0.001 | 129 |
| Role limitations due to emotional problems | −0.427 | 0.002 | 52 | −0.393 | <0.001 | 130 |
| Mental health | −0.526 | <0.001 | 57 | −0.582 | <0.001 | 133 |
| Vitality | −0.448 | <0.001 | 57 | −0.552 | <0.001 | 133 |
| Pain | −0.288 | 0.03 | 57 | −0.266 | 0.002 | 134 |
| General health | −0.522 | <0.001 | 57 | −0.275 | 0.001 | 134 |

Abbreviations: SF-36, Short Form Health 36; MoCa, Montreal Cognitive Assessment; BDI, Beck’s depression inventory II

**Supplement Table 2.** Scores and internal consistency of the Short Form Health 36 questionnaire

| **SF-36** | **Mean** | **SD** | **Ceiling %** | **Floor %** | **Missing %** | Cronbach’s **α** |
| --- | --- | --- | --- | --- | --- | --- |
| **High MoCA ≥ 21** |  |  |  |  |  |  |
| Physical functioning | 47.1 | 29.4 | 3.7 | 5.2 | 0.7 | 0.939 |
| Social functioning | 63.9 | 25.6 | 20.1 | 2.2 | 1.5 | 0.791 |
| Role limitations due to physical problems | 26.9 | 37.5 | 14.0 | 59.7 | 5.1 | 0.868 |
| Role limitations due to emotional problems | 53.6 | 46.3 | 43.8 | 37.7 | 4.4 | 0.908 |
| Emotional well-being | 63 | 17.2 | 0 | 0 | 2.2 | 0.810 |
| Vitality | 45.3 | 17.6 | 0 | 0 | 2.2 | 0.765 |
| Pain | 49.7 | 31 | 17.2 | 5.2 | 1.5 | 0.864 |
| General health | 43 | 24.2 | 0 | 0 | 1.5 | 0.214 |
| **Lower MoCA <21** |  |  |  |  |  |  |
| Physical functioning | 36.2 | 26.5 | 0 | 8.8 | 0 | 0.921 |
| Social functioning | 66.4 | 26.6 | 21.1 | 1.8 | 0 | 0.841 |
| Role limitations due to physical problems | 13.9 | 29.5 | 7.7 | 75.0 | 8.8 | 0.865 |
| Role limitations due to emotional problems | 43.6 | 46.9 | 38.5 | 48.1 | 8.8 | 0.936 |
| Emotional well-being | 58.7 | 16.3 | 0 | 0 | 0 | 0.661 |
| Vitality | 42.6 | 18 | 0 | 1.8 | 0 | 0.698 |
| Pain | 44.5 | 28.2 | 10.5 | 5.3 | 0 | 0.741 |
| General health | 41.8 | 17.1 | 0 | 1.8 | 0 | 0.522 |

Abbreviations: SF-36, Short Form Health 36; MoCa, Montreal Cognitive Assessment

**Supplement Figure 1.** Mediation analyses: Mediation diagrams for SF-36 domains


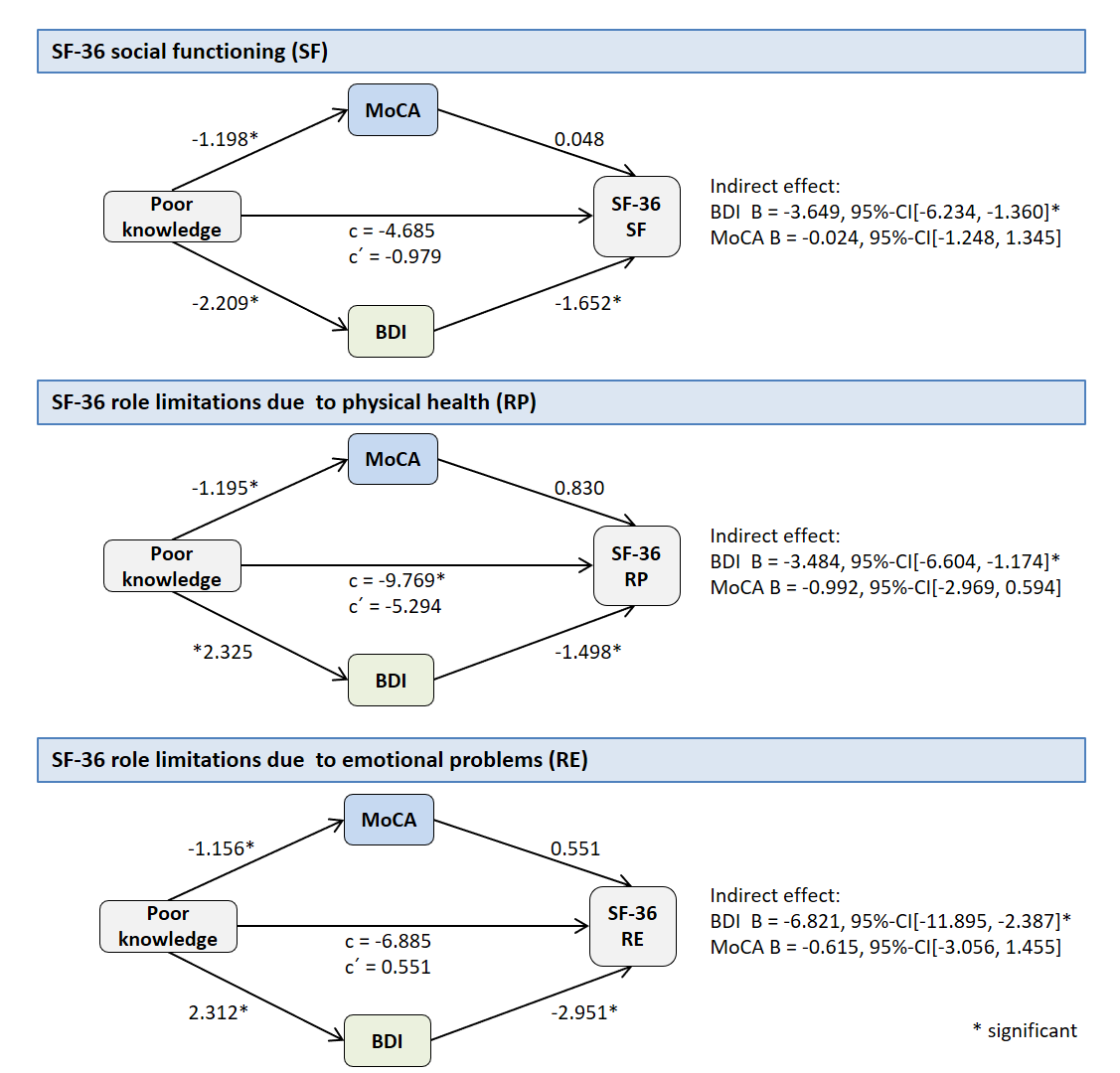


Model summary parameters for SF-social functioning: R^2^= 0.24, MSE = 532.6, p < 0.001

Model summary parameters for SF-physical health: R^2^= 0.18, MSE = 1081.7, p < 0.001

Model summary parameters for SF-emotional problems: R^2^= 0.20, MSE = 1802.4, p < 0.001
